# Pulmonary Embolism with Vertebral Augmentation Procedures

**DOI:** 10.1155/2013/785307

**Published:** 2013-12-09

**Authors:** Swetha Bopparaju, Joseph Varon, Salim Surani

**Affiliations:** ^1^Pulmonary, Critical Care & Sleep Section, Department of Medicine, Baylor College of Medicine, Houston, TX 77030, USA; ^2^Critical Care Services, University General Hospital, Houston, TX 77054, USA; ^3^Acute and Continuing Care, The University of Texas Health Science Center at Houston, USA; ^4^The University of Texas Medical Branch, Houston, TX, USA; ^5^Texas A&M University, Corpus Christi, 1177 West Wheeler Avenue, Suite 1, Aransas Pass, TX 78366, USA

## Abstract

With the prevalence of an aging American population on the rise, osteoporotic vertebral fractures are becoming a common occurrence, resulting in an increase in vertebral augmentation procedures and associated complications such as cement leakage, vertebral compressions, and pulmonary embolism. We describe a patient who presented with respiratory distress three years following kyphoplasty of the lumbar vertebra. Computed tomography (CT) angiogram of the chest confirmed the presence of polymethylmethacrylate (PMMA) cement in the lung fields and pulmonary vessels. We conducted a systematic review of the published literature identifying effective management strategies for the treatment of vertebroplasty-associated pulmonary embolism.

## 1. Introduction

Osteoporotic vertebral fractures are becoming increasingly common in the aging American population. Surgical vertebral augmentation procedures are gaining a high degree of importance and are soon becoming the standard of care to control pain and disability [1, 2]. The beneficial effects of these procedures to provide symptomatic pain relief in patients have been described in clinical trials [1, 2]. However, these augmentation procedures may carry complications involving cement leakage, which range from asymptomatic damage to surrounding tissues and nerves to systemic complications such as compressions and pulmonary embolism [1, 2]. To date, there is a lack of standard diagnostic and therapeutic measures for the management of pulmonary cement embolism. We recently had one such case. In addition, a literature review is presented.

## 2. Case Presentation

**Figure 1:**
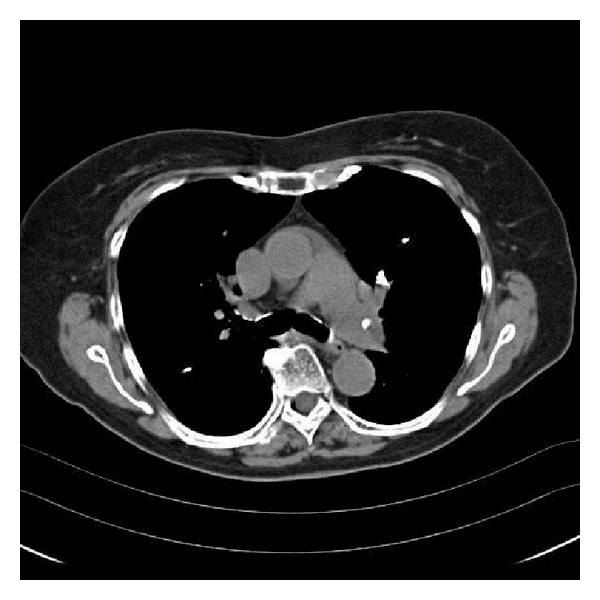
Computed tomography of chest without contrast depicting a clear left pulmonary artery filling defect.

**Figure 2:**
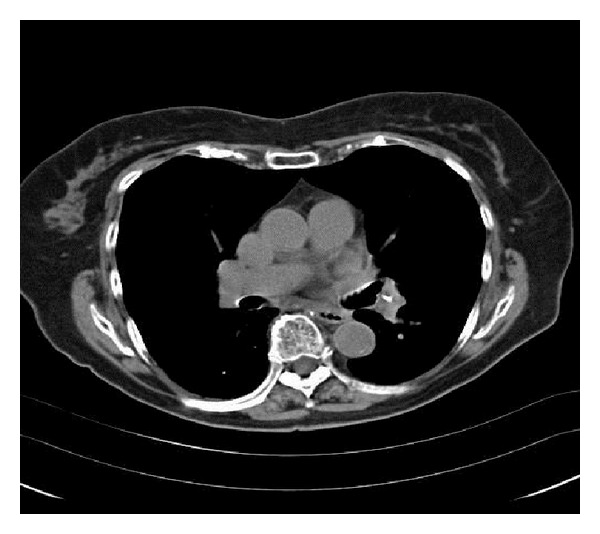
Computed tomography of chest without contrast depicting a filling defect in the branch of left pulmonary artery.

An 82-year-old lady presented to the clinic with complaints of shortness of breath for several months. These symptoms had been worse at the beginning, but her dyspnea on exertion had improved over time. She had a history of osteoporotic vertebral fractures and had undergone kyphoplasty of lumbar vertebra (L4 and L5) three years prior. Blood pressure, hear rate, and respiratory rate were normal and her oxygen saturation was 100% while breathing room air. Her lungs were clear to auscultation and percussion. Cardiac examination was unremarkable as was the rest of her physical exam. A computed tomography (CT) angiogram of the chest revealed radioopaque densities, which were identified in the branch vessels of the pulmonary artery compatible with polymethylmethacrylate (PMMA) embolism (see Figures 1 and 2). She was managed conservatively with a follow-up CT scan three months later revealing no change in PMMA embolism.

## 3. Discussion

Percutaneous vertebral augmentation techniques, such as vertebroplasty, kyphoplasty, and skyphoplasty, are minimally invasive imaging-guided procedures, which are popular treatment protocols for the management of vertebral osteoporotic fractures and osteolytic vertebral tumors (metastasis, myeloma, and hemangiomas). Percutaneous vertebroplasty, the commonest of these procedures, is performed by injecting PMMA cement under high pressure into the vertebral body [3]. Kyphoplasty involves balloon inflation in the vertebral body to create a cavity, which is filled by cement and skyphoplasty involves introduction of plastic tube into the vertebral body [3]. These procedures result in significant pain relief in 70–90% patients, thus accounting for their increasing acceptance [3].

Despite the high rate of success with these procedures, several complications are encountered mostly from cement leakage into the surrounding tissues and systemic circulation [3]. Leakage of PMMA is associated with local side effects, such as spinal canal stenosis, cord compression, and nerve root compression, systemic effects such as pulmonary embolism, and paradoxical cerebral arterial cement embolization through a patent foramen ovale [4]. A higher risk of cement leakage has been noted in vertebroplasty (30%–75%) when compared to kyphoplasty (8–33%) [3]. Interestingly, kyphoplasty has fewer complications since the balloon inflation creates a negative pressure, thereby reducing the risk of intravascular cement leakage while also compacting the osteoporotic bone and sealing the defects and venous blood vessels [5]. PMMA injection consistency is a crucial factor associated with risk of leakage, when using a liquid with low viscosity. A toothpaste-like consistency is ideally recommended, and high pressure, if applied while injecting, would commonly result in cement extravasation [2]. A temperature increase during cement injection may result in rapid polymerization causing damage and lack of bioactivity. New varieties of bioactive composites with lower stiffness properties such as Cortoss and calcium phosphate cement are available but proven to have inferior vertebral strengthening properties when compared to PMMA [6]. Interestingly, higher stiffness in the cement is associated with increased stress on endplates and adjacent vertebrae [7]. Volume and distribution of cement play an important role, with 24% and 30% cement fill required to strengthen vertebral fractures and restore vertebral stiffness, respectively, to improve load-bearing capacity between vertebrae and neural arches [8, 9]. However, high volumes of cement injection may be associated with leakage [10]. Efficient placement of optimal quantity of cement at endplates is performed for better mechanical outcome and strengthens vertebrae while reducing the risk of leakage. Careful monitoring during PMMA injection is imperative with immediate discontinuation of procedure if a leakage or extravasation is suspected [10].

Pulmonary embolism, the most dreaded and, paradoxically, the least symptomatic and diagnosed complication, occurs due to the leakage of cement into the perivertebral veins to enter the pulmonary vascular circulation. Cement, used in these procedures, has a thrombogenic potential leading to pulmonary artery occlusion. PMMA embolisms are detected on X-ray as tubular or branching radiodense lung opacities [10]. Computed tomography (CT) scanning helps to confirm the diagnosis while echocardiogram and pulmonary function tests are helpful additional investigations to evaluate multiple pulmonary emboli, secondary pulmonary arterial pressure elevations, and variations in lung diffusion capacity, respectively [11]. Postoperative chest X-ray is considered justifiable in asymptomatic patients due to high risk of pulmonary embolism ranging from 3.5 to 23%, which is supported by several studies suggesting that followup with chest radiograph, especially in the first 24 hours following percutaneous surgery, is a beneficial measure to detect embolism, even in asymptomatic patients [12].

Pulmonary cement embolisms are usually detected incidentally and less than 1% of patients have presented with clinical symptoms, which explains the lack of clarity in understanding the diagnosis and specific management of this clinical condition [13]. Several studies confirm that patients with cement embolism are commonly asymptomatic or present with symptoms of dyspnea for brief periods of time [14–17]. To our knowledge, there have been four case reports that have documented that percutaneous vertebroplasty resulted in the patients' demise in the published peer-reviewed literature [14–17]. Some clinicians believe that, despite the lack of strong evidence, the use of preinjection venogram is considered to lower the incidence of pulmonary embolism [3].

**Figure 3:**
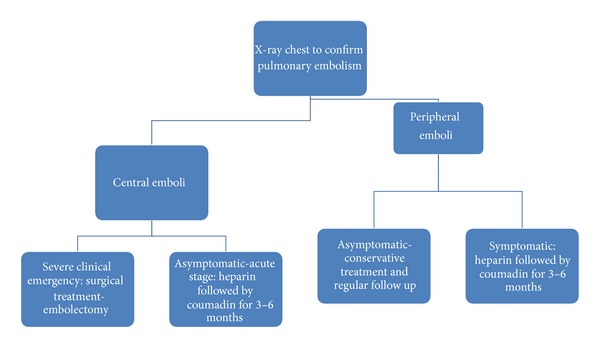
Suggested algorithm for the evaluation and management of patients with cement pulmonary embolism.

The management of these patients is controversial at best. Treatment is recommended to reduce the risk of thrombus formation, pulmonary embolism, pulmonary infarction, and respiratory failure. Some authors have suggested treatment criteria based on the severity of symptoms and location and size of the pulmonary embolism [13, 18]. Among the suggested criteria for treatment, symptomatic patients with peripheral/small emboli should receive conservative treatment with clinical followup and reevaluation, whereas those acute symptomatic patients with central emboli or peripheral emboli should receive treatment with heparin followed by warfarin for 3–6 months [13–18]. In those patients with severe symptoms, severe respiratory failure, and large emboli, aggressive treatment with embolectomy is suggested [18]. Suggested algorithm for the evaluation and management of patients with cement pulmonary embolism is shown in Figure 3.

Treatment with unfractionated heparin followed by warfarin anticoagulation is a viable therapeutic option as it decreases the risk of thrombus formation and allows the foreign material (PMMA cement) to be endothelialized, thus effectively limiting further worsening of the occlusion [12–18]. However, anticoagulation therapy by itself is ineffective in severe cases of pulmonary embolism, since it neither reverses pulmonary infarction and right heart failure symptoms nor improves pulmonary ventilation/perfusion ratio. Aggressive strategy with surgical embolectomy is preferred as an effective approach to remove cement from the pulmonary trunk for immediate resolution of symptoms of cardiorespiratory failure.

## 4. Conclusions

Percutaneous vertebroplasty is a highly effective procedure to treat vertebral compression fractures. The risk of PMMA embolism with a paradoxical silent clinical presentation has limited the outcome of the procedure. Extreme caution is advised to medical personnel while manipulating weakened or tumor-affected vertebrae and ensures that the procedure is performed with critical monitoring and radiological followup for maximum benefit. Further studies for the risks of pulmonary cement embolisms are required, especially with comparison for the different procedures that have not been adequately studied.
